# The prevalence of intimate partner violence in Australia: a national survey

**DOI:** 10.5694/mja2.52660

**Published:** 2025-05-04

**Authors:** Ben Mathews, Kelsey L Hegarty, Harriet L MacMillan, Monica Madzoska, Holly E Erskine, Rosana Pacella, James G Scott, Hannah Thomas, Franziska Meinck, Daryl Higgins, David M Lawrence, Divna Haslam, Sara Roetman, Eva Malacova, Timothy Cubitt

**Affiliations:** ^1^ Queensland University of Technology Brisbane QLD; ^2^ Bloomberg School of Public Health Johns Hopkins University Baltimore United States of America; ^3^ Safer Families Centre, the University of Melbourne Melbourne VIC; ^4^ Family Violence Prevention Centre, the Royal Women's Hospital Melbourne VIC; ^5^ McMaster University Hamilton Canada; ^6^ Curtin University Perth WA; ^7^ The University of Queensland Brisbane QLD; ^8^ Queensland Centre for Mental Health Research Brisbane QLD; ^9^ Institute for Lifecourse Development University of Greenwich London United Kingdom; ^10^ Child Health Research Centre University of Queensland Brisbane QLD; ^11^ Centre for Mental Health Treatment Research and Education, Queensland Centre for Mental Health Research Brisbane QLD; ^12^ University of Edinburgh Edinburgh United Kingdom; ^13^ Institute of Child Protection Studies Australian Catholic University Melbourne VIC; ^14^ Parenting and Family Support Centre the University of Queensland Brisbane QLD; ^15^ QIMR Berghofer Medical Research Institute Brisbane QLD; ^16^ Australian Institute of Criminology Canberra ACT

**Keywords:** Epidemiology, Violence, Women’s rights, Domestic violence, Public health

## Abstract

**Objectives:**

To estimate the prevalence in Australia of intimate partner violence, each intimate partner violence type, and multitype intimate partner violence, overall and by gender, age group, and sexual orientation.

**Study design:**

National survey; Composite Abuse Scale (Revised)—Short Form administered in mobile telephone interviews, as a component of the Australian Child Maltreatment Study.

**Setting:**

Australia, 9 April – 11 October 2021.

**Participants:**

8503 people aged 16 years or older: 3500 aged 16–24 years and about 1000 each aged 25–34, 35–44, 45–54, 55–64, or 65 years or older.

**Main outcome measures:**

Proportions of participants who had ever been in an intimate partner relationship since the age of 16 years (overall, and by gender, age group, and sexual orientation) who reported ever experiencing intimate partner physical, sexual, or psychological violence.

**Results:**

Survey data were available for 8503 eligible participants (14% of eligible persons contacted), of whom 7022 had been in intimate relationships. The prevalence of experiencing any intimate partner violence was 44.8% (95% confidence interval [CI], 43.3–46.2%); physical violence was reported by 29.1% (95% CI, 27.7–30.4%) of participants, sexual violence by 11.7% (95% CI, 10.8–12.7%), and psychological violence by 41.2% (95% CI, 39.8–42.6%). The prevalence of experiencing intimate partner violence was significantly higher among women (48.4%; 95% CI, 46.3–50.4%) than men (40.4%; 95% CI, 38.3–42.5%); the prevalence of physical, sexual, and psychological violence were also higher for women. The proportion of participants of diverse genders who reported experiencing intimate partner violence was high (62 of 88 participants; 69%; 95% CI, 55–83%). The proportion of non‐heterosexual participants who reported experiencing intimate partner violence (70.2%; 95% CI, 65.7–74.7%) was larger than for those of heterosexual orientation (43.1%; 95% CI, 41.6–44.6%). More women (33.7%; 95% CI, 31.7–35.6%) than men (22.7%; 95% CI, 20.9–24.5%) reported multitype intimate partner violence. Larger proportions of participants aged 25–44 years (51.4%; 95% CI, 48.9–53.9%) or 16–24 years (48.4%, 95% CI, 46.1–50.6%) reported experiencing intimate partner violence than of participants aged 45 years or older (39.9%; 95% CI, 37.9–41.9%).

**Conclusions:**

Intimate partner violence is widespread in Australia. Women are significantly more likely than men to experience any intimate partner violence, each type of violence, and multitype intimate partner violence. A comprehensive national prevention policy is needed, and clinicians should be helped with recognising and responding to intimate partner violence.

**The known**: Intimate partner violence is a public health problem with implications for clinical practice and public policy. Information about the prevalence of intimate partner violence in Australia, including differences by gender, age, and sexual orientation, is limited.

**The new**: Intimate partner physical, sexual, and psychological violence is widespread. In our survey, significantly more women reported experiencing each type of violence than men (any intimate partner violence: 48.4% *v* 40.4%). More non‐heterosexual people reported intimate partner violence than heterosexual people (70.2% *v* 43.1%).

**The implications**: The lifetime prevalence of experiencing intimate partner violence is high, especially among women. Improved prevention is needed in the areas of health care, welfare, and justice.

The World Health Organization defines intimate partner violence as “behaviour by an intimate partner or ex‐partner that causes physical, sexual or psychological harm, including physical aggression, sexual coercion, psychological abuse and controlling behaviours.”^1^ The prevalence and severity of experienced intimate partner violence are greater for women than for men.^2^ The United Nations *Declaration on the Elimination of Violence Against Women* ^3^ and target 5.2 of its Sustainable Development Goals^4^ aim to eradicate intimate partner violence.^5^

In Australia, major government initiatives introduced since 2010 aim to improve the understanding and prevention of intimate partner violence.^6^, ^7^ While it affects women, men, and people of diverse genders, national policy acknowledges that most victims are women and that women are more likely than men to experience more severe violence. The *National Plan to End Violence against Women and Children 2022–2032* aims “to end gender‐based violence in one generation.”^6^ Recent reforms have established criminal offences for intimate partner violence within domestic relationships (eg, the New South Wales *Crimes Legislation Amendment (Coercive Control) Act* 2022^8^). This ends centuries of legal doctrine that protected violence by men against women in the private sphere, inaugurating a new era in which gender equality and freedom from violence are fundamental societal principles.

These policy initiatives reflect the consensus that intimate partner violence is a major public health problem with significant and enduring impacts on women's health.^9^, ^10^ It is a leading cause of femicide worldwide,^11^ and it is strongly associated with mental disorders,^12^, ^13^ drug and alcohol problems,^14^, ^15^ and suicide attempts,^12^, ^16^, ^17^ as well as physical conditions such as diabetes, hypertension, and chronic pain.^14^

Intimate partner violence poses challenges for health policy, general practice, specialised clinical care, and the development of practitioner education that is trauma‐informed, violence‐informed, and gender‐informed.^14^, ^17^ Health systems must be able to respond to individual women and women at particular risk.^17^ Reliable and comprehensive population‐based information about its prevalence is fundamental to understanding and preventing intimate partner violence and developing clinical responses.

The global prevalence of intimate partner violence, particularly of violence against women, is concerning;^18^, ^19^, ^20^ the lifetime prevalence among women of physical or sexual intimate partner violence is estimated to be 30%^18^ or 27%.^19^ However, it is increasingly recognised that prevalence studies should use validated measures to assess at least three types of intimate partner violence: physical, sexual, and psychological violence.^12^, ^14^, ^20^, ^21^, ^22^ Rigorously tested instruments for this purpose include the Composite Abuse Scale (CAS)^23^, ^24^ and the Composite Abuse Scale–Revised (short form).^25^

Studies of intimate partner violence in Australia have adopted different approaches. Several have used the CAS (but not to determine population‐wide prevalence), including the Australian Longitudinal Study on Women's Health (ALSWH), which investigated consistency of reporting and health outcomes^26^, ^27^ and associations between intimate partner violence and diverse outcomes.^28^, ^29^ Other research using the CAS has examined intimate partner violence in groups at particular risk,^30^ and intimate partner violence during the coronavirus disease 2019 (COVID‐19) pandemic.^31^

The most reliable estimates of the prevalence of intimate partner violence in Australia have been based on the Australian Bureau of Statistics Personal Safety Survey (PSS).^32^ However, the PSS does not employ a validated scale and covers a restricted range of behaviours. For example, “intimate partners” includes only partners who live together; “sexual violence” excludes unwanted sexual touching and is limited to forced or attempted forced sexual activity against the person's will; and physical and emotional violence items are confined to actions in which the perpetrator intended to cause harm. In 2021–22, the PSS found that the lifetime prevalence of physical or sexual abuse was 23% for women and 7.3% for men, of emotional abuse 23% for women and 14% for men, and of economic abuse 16% for women and 7.8% for men.^32^

Important gaps in our knowledge about the prevalence and nature of intimate partner violence remain, both in Australia and overseas.^12^, ^14^, ^19^, ^33^, ^34^ No Australian survey with a nationally representative sample and using a validated instrument has estimated the prevalence of a broad spectrum of intimate partner violence, including multitype intimate partner violence, across all intimate relationships. Further, little is known about differences in prevalence by gender, age group, or sexual orientation. A survey of a large nationally representative sample that captured the lifetime experiences of diverse types of intimate partner violence could help close these knowledge gaps.

The primary aim of our study was to estimate the national prevalence of intimate partner violence in Australia by surveying a nationally representative sample of people, using a psychometrically validated instrument. We also estimated the national prevalence of multitype intimate partner violence and of each specific intimate partner violence type, and examined differences by gender, age group, and sexual orientation.

## Methods

Our survey was undertaken as part of the Australian Child Maltreatment Study (ACMS). The aims of the ACMS were to estimate the prevalence of child maltreatment and of associated mental disorders, health risk behaviours, and burden of disease.^35^ The ACMS also provided an opportunity to explore intimate partner violence. As detailed elsewhere,^36^ 8503 participants aged 16 years or older were recruited for the ACMS mobile telephone survey during 9 April – 11 October 2021 by a professional survey company, the Social Research Centre (https://srcentre.com.au) using random mobile phone digit dial technology and advance text messaging. The demographic characteristics of the participant group were similar to those of the Australian population in 2016^37^ with respect to gender, Indigenous status, region (metropolitan, regional/rural) and remoteness category of residence,^38^ and marital status, but larger proportions of participants were born in Australia, lived in areas of higher socio‐economic status (Index of Relative Socio‐economic Advantage and Disadvantage, IRSAD^39^), had tertiary qualifications, or had income exceeding $1250 per week. Population weights were derived to adjust for these differences (Supporting Information, table 1). We report our study according to the Checklist for Reporting of Survey Studies (CROSS).^40^

### Measures

The ACMS administered the Composite Abuse Scale (Revised)–Short Form (CAS_R_‐SF), a validated and reliable measure of intimate partner violence.^25^ The CAS_R_‐SF comprises fifteen behaviour‐specific questions (yes/no responses) about experiences of intimate partner violence (five questions about physical violence; two about sexual violence; and eight about psychological violence). The items were introduced with a preamble that asked the participant to indicate whether any intimate partner had ever inflicted any of the acts on them.

Sex and gender were assessed with the question, “How would you define your gender?” Responses were coded using a list of fourteen options, including “female/woman” and “male/man”. For this report, we categorised participants who did not select either of these options as being of diverse gender.

### Procedures

Study procedures have been reported elsewhere.^36^ In brief, the 8503 participants included 3500 participants aged 16–24 years, and about 1000 participants each aged 25–34, 35–44, 45–54, 55–64, or 65 years or older. The sample, including the oversampled 16–24‐year‐old age group, met the research aims of both the ACMS and our survey. Data from the computer‐assisted telephone interview (CATI) software platform were imported into SAS 9.4; data cleaning was undertaken by authors DML and MM.

### Statistical analysis

We calculated the prevalence of any intimate partner violence for the entire participant group, and by gender, age group, and sexual orientation. For each intimate partner violence type, we adopted methods congruent with the instrument^25^ and other studies,^30^ generating estimated prevalence based on positive endorsement of any of the screeners. For the prevalence of multitype intimate partner violence, we aggregated data for people who experienced two or all three of the three intimate partner violence types. We calculated prevalence rates at the whole of population level for people who had ever been in an intimate relationship since the age of 16 years, including all whose marital status was married, separated, divorced, or living together but not married or widowed; it also included those who were single or never married, but who had ever “been in an adult intimate relationship”, defined as being in a romantic relationship when aged 16 years or older.

Survey‐weighted data were summarised as numbers and proportions with 95% confidence intervals (CIs) calculated using the Taylor series method. We compared each type of intimate partner violence by gender, sexual orientation, and age group; statistical significance of between group differences was defined as non‐overlap of the 95% CIs. We also compared prevalence estimates for participants aged 25–44 years and those aged 45 years or older using the Rao–Scott χ^2^ test for survey data; more than 90% of participants in these two age groups had had intimate partners, but 42% of people aged 16–24 years had not. Missing responses were treated as “no” responses. All analyses were conducted in SAS 9.4; graphs were prepared in Stata 17. All analyses were checked independently by two authors, by random spot checking of the SAS coding and replicating analyses in SPSS 28.

### Ethics approval

The ACMS, including the separately funded intimate partner violence component, was approved by the Queensland University of Technology Human Research Ethics Committee (#1900000477). Each survey participant provided verbal informed consent to participation.

## Results

Of the 404 180 people we attempted to contact by phone, 210 373 would have been eligible to participate in our survey; contact was made with 60 803 eligible persons, of whom 8503 completed the ACMS survey (Supporting Information, table 1). The response rate among eligible candidates contacted by phone was 14.0%; based on the total number of eligible candidates, including those not contacted, it was 4.0%. Potential participation bias, as indicated by number of calls required to complete the survey, was deemed to be minor.^36^

Of the 8503 respondents, 21 (0.25%) did not report whether they had ever been in intimate partner relationships and were not asked questions about intimate partner violence. Of the remaining 8482 participants, 8357 (98.5%) identified as female/woman or male/man; 7022 (unweighted proportion, 82.6%; weighted proportion, 90.9%) had had intimate partners at some point since age sixteen years, including 2201 of 3493 people aged 16–24 years (weighted proportion, 56.9%; women, 59.7%; men, 54.2%; diverse genders, 54.6%) 1868 of 1993 people aged 25–44 years (92.9%; women, 96.0%; men, 89.9%; diverse genders, 92.9%), and 2953 of 2996 people aged 45 years or older (98.5%; women, 98.9%; men, 98.1%) (Supporting Information, table 2).

### Prevalence of intimate partner violence, by gender and sexual orientation

All proportions reported from this point are weighted proportions. A total of 3170 participants reported ever experiencing intimate partner violence since the age of 16 years (44.8%; 95% CI, 43.3–46.2%); the proportion was significantly larger for women (48.4%; 95% CI, 46.3–50.4%) than men (40.4%; 95% CI, 38.3–42.5%). Sixty‐two of 88 participants of diverse genders reported experiencing intimate partner violence (69%; 95% CI, 55–83%) (Box 1).

Box 1 Lifetime experience of any intimate partner violence among 7022 respondents with intimate partners at any time since age 16 years, overall and by age group and gender*

**Table:** 

Age group	Respondents	Number reporting experience	Proportion (95% CI)^†^
All respondents	7022	3170	44.8% (43.3–46.2%)
Women	3558	1763	48.4% (46.3–50.4%)
Men	3376	1345	40.4% (38.3–42.5%)
Diverse genders	88	62	69% (55–83%)
16–24 years	2201	1056	48.4% (46.1–50.6%)
Women	1099	576	52.5% (49.3–55.7%)
Men	1048	442	42.8% (39.5–46.1%)
Diverse genders	54	38	71% (58–83%)
25–44 years	1868	928	51.4% (48.9–53.9%)
Women	945	499	53.8% (50.3–57.3%)
Men	903	414	48.5% (44.8–52.1%)
45 years or older	2953	1186	39.9% (37.9–41.9%)
Women	1514	688	44.3% (41.5–47.1%)
Men	1425	489	34.6% (31.9–37.4%)

CI = confidence interval.

* Rao Scott χ^2^ test of association between gender or age group and lifetime experience of intimate partner violence: each *P* < 0.001. The numbers and proportions of respondents who experienced the three types of intimate partner violence are reported in the Supporting Information, table 3; the numbers and proportions did not provide responses for specific items are reported in the Supporting Information, table 4.

† Weighted by age group, sex, Indigenous status, country of birth (Australia or overseas), highest educational level, and residential socio‐economic status (Relative Socio‐economic Advantage and Disadvantage quintile).

The proportion of participants who reported ever experiencing intimate partner violence was significantly larger for those of non‐heterosexual orientation (70.2%; 95% CI, 65.7–74.7%) than for participants of heterosexual orientation (43.1%; 95% CI, 41.6–44.6%) (Box 2).

Box 2 Lifetime experience of any intimate partner violence among 7022 respondents with intimate partners at any time since age 16 years, overall and by age group and sexual orientation*

**Table:** 

Age group	Respondents	Number reporting experience	Proportion (95% CI)^†^
All respondents	7022	3170	44.8% (43.3–46.2%)
Heterosexual	6214	2650	43.1% (41.6–44.6%)
Diverse sexualities	730	494	70.2% (65.7–74.7%)
Do not know/no response	78	—	—
16–24 years	2201	1056	48.4% (46.1–50.6%)
Heterosexual	1759	770	44.5% (41.9–47.0%)
Diverse sexualities	433	282	64.1% (59.1–69.1%)
25–44 years	1868	928	51.4% (48.9–53.9%)
Heterosexual	1653	785	49.6% (47.0–52.3%)
Diverse sexualities	189	136	71.9% (64.8–79.0%)
45 years or older	2953	1186	39.9% (37.9–41.9%)
Heterosexual	2802	1095	38.9% (36.9–41.0%)
Diverse sexualities	108	76	72.7% (62.9–82.4%)

CI = confidence interval.

* Rao Scott χ^2^ test of association between sexual orientation and lifetime experience of intimate partner violence, both overall and for each age group: each *P* < 0.001.

† Weighted by age group, sex, Indigenous status, country of birth (Australia or overseas), highest educational level, and residential socio‐economic status (Relative Socio‐economic Advantage and Disadvantage quintile).

### Prevalence of types of intimate partner violence, by gender

A total of 1889 participants reported intimate partner physical violence (29.1%; 95% CI, 27.7–30.4%); the proportion was significantly larger for women (32.3%; 95% CI, 30.3–34.2%) than for men (25.4%; 95% CI, 23.5–27.2%) (Box 3, Box 4). The prevalence of four of the five types of physical violence were higher for women, including being choked (women: 11.6%, 95% CI, 10.3–12.9%; men: 3.8%: 95% CI, 3.0–4.6%) and actual or threatened harm by a knife, gun, or weapon (women: 10.1%, 95% CI, 8.8–11.4%; men: 7.5%, 95% CI, 6.3–8.6%) (Box 5; Supporting Information, table 4).

Box 3 Lifetime experience of intimate partner violence types among 7022 respondents with intimate partners at any time since age 16 years, overall and by age group and gender*

**Table:** 

		Physical violence		Sexual violence		Psychological violence	
Age group	Respondents	Number	Proportion (95% CI)^†^	Number	Proportion (95% CI)^†^	Number	Proportion (95% CI)^†^
All respondents	7022	1889	29.1% (27.7–30.4%)	925	11.7% (10.8–12.7%)	2897	41.2% (39.8–42.6%)
Women	3558	1068	32.3% (30.3–34.2%)	711	18.2% (16.6–19.8%)	1631	45.1% (43.1–47.1%)
Men	3376	788	25.4% (23.5–27.2%)	175	4.0% (3.3–4.8%)	1216	36.6% (34.6–38.6%)
Diverse genders	88	33	37% (23–51%)	39	42% (28–56%)	50	56% (42–71%)
16–24 years	2201	530	25.2% (23.2–27.2%)	401	18.2% (16.5–20.0%)	964	44.2% (42.0–46.5%)
Women	1099	287	26.8% (24.0–29.6%)	286	25.8% (23.0–28.6%)	529	48.4% (45.2–51.6%)
Men	1048	222	22.6% (19.7–25.5%)	89	8.5% (6.5–10.4%)	405	39.3% (36.0–42.5%)
25–44 years	1868	564	33.0% (30.6–35.4%)	252	13.9% (12.2–15.7%)	866	48.2% (45.7–50.7%)
Women	945	301	34.8% (31.4–38.2%)	185	20.8% (17.9–23.7%)	474	51.5% (47.9–55.0%)
Men	903	257	31.0% (27.6–34.4%)	57	5.9% (4.3–7.5%)	378	44.4% (40.7–48.0%)
45 years or older	2953	795	27.1% (25.3–28.9%)	272	9.3% (8.1–10.5%)	1067	36.2% (34.2–38.1%)
Women	1514	480	31.5% (28.8–34.1%)	240	15.5% (13.4–17.5%)	628	40.7% (37.9–43.4%)
Men	1425	309	22.0% (19.6–24.4%)	29	2.1% (1.3–3.0%)	433	31.0% (28.3–33.7%)

CI = confidence interval.

* Rao Scott χ^2^ test of association between gender and each intimate partner violence type: each *P* < 0.001.

† Weighted by age group, sex, Indigenous status, country of birth (Australia or overseas), highest educational level, and residential socio‐economic status (Relative Socio‐economic Advantage and Disadvantage quintile).

Box 4 Lifetime experience of intimate partner violence types among 7022 respondents with intimate partners at any time since age sixteen, by gender*

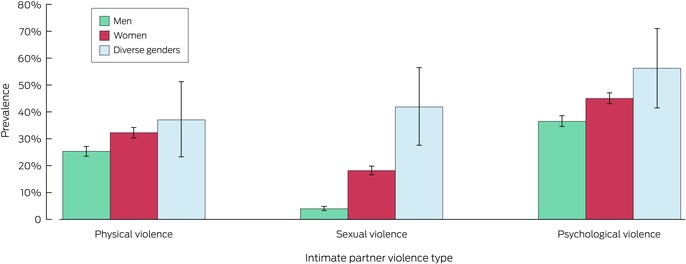

* The data for this graph are included in Box 3. Proportions are weighted by age group, sex, Indigenous status, country of birth (Australia or overseas), highest educational level, and residential socio‐economic status (Relative Socio‐economic Advantage and Disadvantage quintile).

Box 5 Lifetime experience of specific forms of intimate partner violence types among 7022 respondents with intimate partners at any time since age 16 years, by gender*

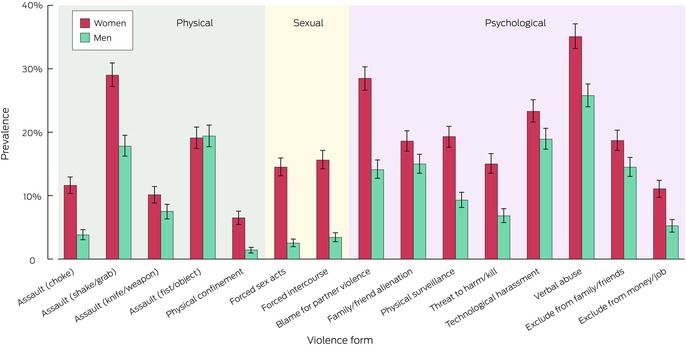

* The data for this graph are included in Supporting Information, tables 5 to 7. Proportions are weighted by age group, sex, Indigenous status, country of birth (Australia or overseas), highest educational level, and residential socio‐economic status (Relative Socio‐economic Advantage and Disadvantage quintile).

A total of 925 participants reported intimate partner sexual violence (11.7%; 95% CI, 10.8–12.7%); the proportion was significantly larger for women (18.2%; 95% CI, 16.6–19.8%) than for men (4.0%; 95% CI, 3.3–4.8%) (Box 3, Box 4). The prevalence of both types of sexual violence were higher for women: forced sex or attempted forced sex (women: 15.6%; 95% CI, 14.2–17.1%; men: 3.4%, 95% CI; 2.7–4.1%), and being made to perform sex acts they did not want to (women: 14.5%; 95% CI, 13.1–15.9%; men: 2.5%; 95% CI, 1.9–3.1%) (Box 5; Supporting Information, table 5).

A total of 2897 participants reported intimate partner psychological violence (41.2%; 95% CI, 39.8–42.6%); the proportion was significantly larger for women (45.1%; 95% CI, 43.1–47.1%) than for men (36.6%; 95% CI, 34.6–38.6%) (Box 3, Box 4). The prevalence of all eight types of psychological violence were higher for women (Box 5; Supporting Information, table 6).

### Prevalence of intimate partner violence, by age

A significantly larger proportion of participants aged 25–44 years reported ever experiencing intimate partner violence (51.4%; 95% CI, 48.9–53.9%) than of participants aged 45 years or older (39.9%; 95% CI, 37.9–41.9%). A total of 1056 participants aged 16–24 years reported any intimate partner violence (48.4%, 95% CI, 46.1–50.6%) (Box 1).

### Prevalence of multitype intimate partner violence

A total of 1925 participants reported two or more types of intimate partner violence (28.6%; 95% CI, 27.2–29.9%); the proportion was significantly larger for women (33.7%; 95% CI, 31.7–35.6%) than for men (22.7%; 95% CI, 20.9–24.5%) (Box 6). The proportion of respondents who reported all three intimate partner violence types was also significantly larger for women (13.6%; 95% CI, 12.1–15.0) than for men (2.9%; 95% CI, 2.2–3.6) and by age group (Box 7). A total of 2185 participants at least one of physical or sexual violence (32.0%; 95% CI, 30.7‐33.4%); the proportion was significantly larger for women (36.7%; 95% CI, 34.8–38.7%) than men (26.5%; 95% CI, 24.6–28.3%) (Supporting Information, table 8).

Box 6 Lifetime experience of multitype intimate partner violence among 7022 respondents with intimate partners at any time since age 16 years, overall and by age group and gender*

**Table:** 

		Multitype intimate partner violence		Two types		Three types	
Age group	Respondents	Number	Proportion (95% CI)^†^	Number	Proportion (95% CI)^†^	Number	Proportion (95% CI)^†^
All respondents	7022	1925	28.6% (27.2–29.9%)	1309	19.9% (18.7–21.1%)	616	8.6% (7.8–9.5%)
Women	3558	1169	33.7% (31.7–35.6%)	691	20.1% (18.5–21.7%)	478	13.6% (12.1–15.0%)
Men	3376	718	22.7% (20.9–24.5%)	602	19.8% (18.1–21.5%)	116	2.9% (2.2–3.6%)
Diverse genders	88	38	41% (27–56%)	16	16% (6.5–26%)	22	25% (12–38%)
16–24 years	2201	606	28.3% (26.2–30.4%)	373	17.4% (15.6–19.1%)	233	10.9% (9.5–12.4%)
Women	1099	362	33.2% (30.2–36.3%)	198	18.0% (15.6–20.5%)	164	15.2% (12.9–17.5%)
Men	1048	220	22.1% (19.3–24.9%)	166	16.7% (14.1–19.2%)	54	5.4% (3.8–7.1%)
Diverse genders	54	24	46% (32–60%)	9	17% (6.0–28%)	15	29% (16–42%)
25–44 years	1868	583	33.6% (31.2–36.0%)	412	23.5% (21.3–25.7%)	171	10.1% (8.5–11.7%)
Women	945	335	38.0% (34.5–41.4%)	209	22.7% (19.8–25.7%)	126	15.3% (12.6–17.9%)
Men	903	238	28.6% (25.3–32.0%)	198	24.5% (21.3–27.7%)	40	4.1% (2.8–5.5%)
45 years or older	2953	736	25.3% (23.6–27.1%)	524	18.0% (16.4–19.5%)	212	7.3% (6.3–8.4%)
Women	1514	472	31.0% (28.4–33.7%)	284	18.8% (16.5–21.0%)	188	12.3% (10.4–14.1%)
Men	1425	260	18.8% (16.6–21.1%)	238	17.1% (15.0–19.3%)	22	1.7% (0.9–2.4%)

CI = confidence interval.

* Rao‐Scott χ^2^ test of association between gender or age group and multitype intimate partner violence: each *P* < 0.001. The experience of each combination of intimate sexual partner violence type, extrapolated to the entire Australian population, is depicted in the Supporting Information, figure 1.

† Weighted by age group, sex, Indigenous status, country of birth (Australia or overseas), highest educational level, and residential socio‐economic status (Relative Socio‐economic Advantage and Disadvantage quintile).

Box 7 Lifetime experience of all three types of intimate partner violence among 7022 respondents with intimate partners at any time since age 16 years, overall and by age group and gender*

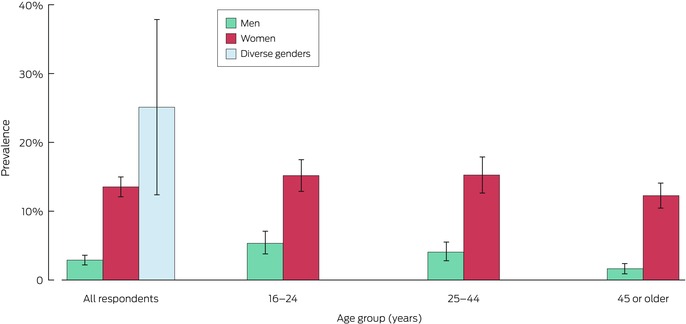

* The data for this graph are included in Box 6. Proportions are weighted by age group, sex, Indigenous status, country of birth (Australia or overseas), highest educational level, and residential socio‐economic status (Relative Socio‐economic Advantage and Disadvantage quintile).

## Discussion

We report national prevalence estimates for the lifetime experience of intimate partner violence, both overall and by type: physical, sexual, and psychological. Using a validated instrument and a large nationally representative sample, we found that 44.8% of respondents aged 16 years or older had experienced intimate partner violence. The comprehensive survey covered experiences during all intimate partner relationships, and a broad range of types and forms of intimate partner violence. We found that the prevalence of experiencing physical, sexual, and psychological violence among women was higher than reported by an Australian survey that captured information only on violence by cohabiting partners and that excluded some types of violence.^32^ Further, we found the prevalence of experience of physical or sexual violence among women (36.7%) was higher than published estimates of its worldwide prevalence (30.0%,^18^ 27.0%^19^).

The prevalence of intimate partner violence experienced by women is particularly concerning. A significantly larger proportion of women than men reported experiencing any intimate partner violence, both overall (48.4% *v* 40.4%) and for all three types. Physical violence was reported by 32.3% of women, sexual violence by 18.2%, and psychological violence by 45.1%. Intimate partner violence clearly remains a major public health problem that requires more effective and comprehensive public health solutions.

Substantial proportions of men reported physical (25.4%) and psychological intimate partner violence (36.6%). Physical violence against men by women can involve retaliatory or defensive responses to intimate partner violence by their partners, and can be less severe than violence inflicted by men.^41^ It is possible that much physical violence by women against men is “situational couple violence” rather than the ongoing “intimate terrorism” that comprises a substantial proportion of intimate partner violence against women.^42^ The prevalence of specific forms of physical violence in our survey indicated that men experienced severe physical violence less frequently than women (eg, choking: men, 3.8% *v* women, 11.6%; violence with a knife, gun, or weapon: men, 7.5% *v* women, 10.1%). Being hit with a fist or object or being kicked or bitten was reported by 19.4% of male respondents, and being shaken, pushed, grabbed, or thrown by 17.8%; these forms of violence could be inflicted in the context of situational couple violence. The experiences of men should not be dismissed, but the prevalence of any intimate partner violence, each intimate partner violence type, multitype intimate partner violence, and fourteen of the fifteen specific violence forms was significantly higher for women than men (Box 5).

Our findings regarding multitype intimate partner violence indicate that women experience more varied intimate partner violence than men: 33.7% of women experienced two or three types of intimate partner violence, compared with 22.7% of men, and 13.6% of women experienced all three types, compared with 2.9% of men (Box 6). Regardless of whether the different violence types were inflicted by one or by several perpetrators, this cumulative experience of violence is concerning, given the likelihood of especially poor health consequences for people who experience multiple types of intimate partner violence.^33^

Analysis by age group suggests that the prevalence of intimate partner violence has not declined. Cross‐sectional surveys of lifetime intimate partner violence typically find that the reported prevalence is higher among younger participants, despite their having had less time to be in relationships and the expectation that cumulative prevalence would increase with time.^41^ Younger people may be particularly exposed to intimate partner violence, and some older participants may not recall less serious or isolated incidents.^43^ Whatever the impact of these factors, we found that the prevalence of intimate partner violence among participants aged 25–44 years was significantly higher (51.4%) than for those aged 45 years or older (39.9%); and it was significantly higher for women aged 25–44 years (53.8%) than for women aged 45 years or older (44.3%). The prevalence among women aged 16–24 years (52.5%), was similar to that for women aged 25–44 years. Among all participants aged 16–24 years, 48.4% had experienced intimate partner violence, including physical violence (25.2%) and sexual violence (18.2%). These age group findings, especially those for women, indicate that intimate partner violence remains a major problem in Australian society.

Our study is one of the few to examine lifetime intimate partner violence experienced by non‐heterosexual people.^34^ Its prevalence was higher among non‐heterosexual (70.2%) than heterosexual participants (43.1%). The small number of non‐heterosexual participants who had been in intimate relationships (494 people) constrains interpretation of this finding, and further research is needed. Similarly, the prevalence of intimate partner violence experienced by people of diverse genders (69%) was higher than for women, but the small number of respondents (88 people) again means that further research is required before drawing conclusions.

We collected data from a large, nationally representative sample of Australians, obtaining rigorous population‐wide data about the lifetime prevalence of intimate partner violence. We used a psychometrically validated instrument, ensuring robust and comprehensive measurement of multiple forms of intimate partner violence, capturing their full extent without excluding important types of violence. We explicitly assessed psychological violence, an important and neglected form of intimate partner violence.^2^, ^19^, ^44^ We captured intimate partner violence inflicted by all intimate partners, not only those with whom the victim lived, generating a more complete picture of intimate partner violence in Australia.

### Limitations

The retrospective design of our study may have introduced recall bias,^42^ leading to conservative prevalence estimates. Some experiences may have been cognitively reframed by individual participants as normal, also reducing prevalence. We did not assess the frequency or intensity of intimate partner violence; our primary aim was to estimate lifetime prevalence of intimate partner violence, but the prevalence of specific forms could provide insights about severity. The COVID‐19 pandemic may have influenced the prevalence of some forms of intimate partner violence,^31^ but any association with the onset or intensification of existing intimate partner violence is unlikely to have substantially affected lifetime prevalence. As our purpose was to provide population‐wide prevalence estimates, we did not assess prevalence by ethnic background or Indigenous status; in any case, small participant numbers in these categories precluded such analyses. Further analyses by Indigenous status should be considered only in studies by Indigenous research teams, and designed by, with, and for Indigenous communities. Finally, we did not take an intersectional approach to assessing the relationship between multiple social categories and intimate partner violence.

### Conclusion

Intimate partner violence is widespread in Australia. The prevalence of all types of intimate partner violence is significantly higher among women than men. National intimate partner violence prevention strategies have not yet achieved their objectives; they should be strengthened by a comprehensive, long term approach to responding to risk factors, including social and economic conditions, together with long standing societal and structural discrimination against women, prejudicial social norms, and diverse individual risk factors for violence by men.^9^ Health systems and clinical practice can also contribute to primary prevention and early intervention by assisting individuals and people in groups at particular risk of violence. Practitioner education and system change is required to support violence‐informed, trauma‐informed, and gender‐informed patient care.^9^, ^10^, ^17^ Intimate partner violence should be further investigated, including its chronicity and contexts, to monitor trends and identify areas of specific need.

## Open access

Open access publishing facilitated by Queensland University of Technology, as part of the Wiley – Queensland University of Technology agreement via the Council of Australian University Librarians.

## Competing interests

No relevant disclosures.

## Data sharing

Final data sets will be stored on the Australian Data Archive and made available in January 2026 after an embargo period.

## Supporting information


Supplementary methods and results
