# Supplementary material for: The prevalence of intimate partner violence in Australia: a national survey
Source: Med J Aust. 2025 May 4;222(9):440–8. doi: 10.5694/mja2.52660 (PMC12088308; doi:10.5694/mja2.52660)
Supplement: Supplementary file 1 — Supplementary methods and results [file MJA2-222-440-s001.pdf]

The Medical Journal of Australia

Supporting Information

Supplementary methods and results

This appendix was part of the submitted manuscript and has been peer reviewed. It is posted as supplied by the authors.

Appendix to: Mathews B, Hegarty KL, MacMillan HL, et al. The prevalence of intimate partner violence in Australia: a national survey. *Med J Aust* 2025; doi: 10.5694/mja2.52660.

Supplementary methods

Table 1. Demographic characteristics of the 8503 participants in the Australian Child Maltreatment Study survey, 9 April – 11 October 2021, and of Australians aged 16 years or older, according to the 2016 Australian census*

	Australian Child Maltreatment Study participants			
**Characteristic**	**Number**	**Unweightedproportion**	**Weighted proportion†**	**2016 census‡**
Gender				
Men	4195	49.3%	48.1%	48.8%
Women	4182	49.2%	50.9%	51.2%
Non-binary/other	126	1.5%	1.0%	NA
Age group (years)				
16–24	3500	41.1%	13.6%	13.6%
25–34	1000	11.8%	18.2%	18.2%
35–44	1000	11.8%	17.0%	17.0%
45–54	1002	11.8%	15.7%	15.7%
55–64	1001	11.8%	14.5%	14.5%
65 or more	1000	11.8%	20.9%	20.9%
Indigenous status				
Aboriginal or Torres Strait Islander	290	3.4%	2.7%	2.7%
Non-Indigenous	8176	96.2%	96.8%	91.5%
Not stated	37	0.4%	0.5%	6.2%
Marital status				
Single/never married	4046	47.7%	29.8%	27.4%
Living together but not married	918	10.8%	11.0%	15.3%
Married	2715	32.0%	43.9%	32.0%
Separated/divorced/widowed	803	9.4%	15.3%	14.6%
Residence: region (1)				
Metropolitan	5798	68.2%	64.4%	67.0%
Regional/rural	2705	31.8%	35.6%	33.0%
Residence: remoteness (1)				
Major cities	6247	73.5%	69.6%	72.1%
Inner regional	1471	17.3%	19.1%	18.0%
Outer regional	658	7.7%	9.5%	8.1%
Remote	83	1.0%	1.2%	1.0%
Very remote	44	0.5%	0.6%	0.8%
Birthplace: participant				
Australia	6347	74.6%	65.9%	65.9%
Overseas	2146	25.3%	34.0%	28.3%
Not stated	10	0.1%	0.1%	7.1%
Birthplace: parents				
Both in Australia	4362	51.3%	48.9%	49.0%
One in Australia	1351	15.9%	10.6%	10.6%
Both overseas	2762	32.5%	40.2%	40.4%
Not known	28	0.3%	0.3%	< 0.1%

	Australian Child Maltreatment Study participants			
**Characteristic**	**Number**	**Unweightedproportion**	**Weighted proportion†**	**2016 census‡**
Highest level of educational attainment				
Postgraduate degree	1100	12.9%	8.1%	8.0%
Undergraduate degree	1859	21.9%	17.9%	17.7%
College certificate/diploma	1385	16.3%	19.6%	19.4%
Year 12	2273	26.8%	20.6%	21.2%
Trade certificate	692	8.1%	13.5%	13.4%
Year 10	1091	12.8%	18.3%	18.1%
Year 9 or below	78	0.9%	2.1%	2.1%
Employment status				
Employed fulltime	3601	42.5%	43.1%	39.1%
Employed part-time	2372	27.9%	21.3%	20.3%
Unemployed	724	8.5%	7.4%	4.5%
Not in the labour force	1779	21.0%	28.2%	36.0%
Index of Relative Socio-economic Advantage and Disadvantage (2)				
1 (lowest)	1086	12.8%	15.6%	15.6%
2	1180	13.9%	15.9%	15.9%
3	1497	17.6%	19.0%	19.0%
4	1938	22.8%	20.8%	20.8%
5 (highest)	2802	33.0%	28.7%	28.7%
Individual income (weekly)				
Below $500	2316	27.2%	25.1%	45.6%
$500–1249	2158	25.4%	24.0%	32.0%
$1250 or more	2496	29.4%	32.6%	22.4%
Not stated	1533	18.0%	18.3%	NA

NA = not applicable (not an option in 2016 census).

* Source: Reference (3), which also reports all characteristics by age group.

† Weighted by gender, age group, Indigenous status, country of birth, highest education level, and postcode-level Index of Relative Socio-economic Advantage and Disadvantage.

‡ Source: 2016 Australian Bureau of Statistics Census of Population and Housing (using TableBuilder Basic), as reported in reference (3).

Supplementary results

Table 2. Demographic characteristics of 8482 participants in the Australian Child Maltreatment Study survey, 9 April – 11 October 2021, by whether they had ever been in an intimate partnership since age 16 years*

	Ever had a partner		Never had a partner	
**Age group**	**Number**	**Weighted proportion†**	**Number**	**Weighted proportion†**
**All respondents**	**7022**	**90.9% (90.3-91.5%)**	**1460**	**8.8% (8.2-9.5%)**
Women	3558	92.8% (92.1-93.5%)	615	7.0% (6.3-7.7%)
Men	3376	89.0% (99.0-90.1%)	808	10.6% (9.6-11.6%)
Diverse genders	88	82.1% (74.9-89.2%)	37	17.1% (10.1-24.1%)
**16-24 years**	**2201**	**56.9% (55.0-58.7%)**	**1292**	**42.9% (41.0-44.8%)**
Women	1099	59.7% (57.0-62.4%)	559	40.1% (37.4-42.8%)
Men	1048	54.2% (51.6-56.8%)	697	45.6% (42.9-48.2%)
Diverse genders	54	54.6% (43.1-66.1%)	36	45.4% (33.9-56.9%)
**25-44 years**	**1868**	**92.9% (91.7-94.2%)**	**125**	**6.7% (5.4-7.9%)**
Women	945	96.0% (94.7-97.3%)	39	3.8% (2.5-5.1%)
Men	903	89.9% (87.7-92.1%)	85	9.6% (7.4-11.7%)
Diverse genders	20	92.9% (82.5-100.0%)	†	‡
**45 years and over§**	**2953**	**98.5% (98.1-98.9%)**	**43**	**1.3% (0.9-1.7%)**
Women	1514	98.9% (98.4-99.4%)	17	0.9% (0.4-1.4%)
Men	1425	98.1% (97.3-98.8%)	26	1.7% (1.0-2.4%)
**Region (1)**				
Metropolitan	4712	89.8% (88.9-90.6%)	1068	9.9% (9.1-10.7%)
Regional/Rural	2310	93.0% (92.0-93.9%)	392	6.9% (6.0-7.8%)
**Remoteness (2)**				
Major cities	5091	89.9% (89.1-90.7%)	1138	9.8% (9.0-10.6%)
Inner regional	1269	93.6% (92.5-94.8%)	200	6.1% (5.0-7.2%)
Outer regional	555	92.4% (90.4-94.5%	102	7.5% (5.5-9.5%)
Remote or very remote	107	92.0% (86.4-97.5%)	20	8.0% (2.5-13.6%)
**IRSAD quintile (3)**				
1 (lowest)	869	90.8% (89.1-92.4%)	216	9.2% (7.6-10.8%)
2	984	91.7% (90.2-93.2%)	194	8.1% (6.6-9.6%)
3	1244	91.7% (90.2-93.1%)	251	8.2% (6.8-9.6%)
4	1589	90.3% (88.9-91.7%)	342	9.4% (8.0-10.8%)
5 (highest)	2336	90.4% (89.3-91.5%)	457	9.1% (8.0-10.1%)
**Marital status**				
Single/never married	2586	70.3% (68.4-72.1%)	1460	29.7% (27.9-31.6%)
Living together but not married	918	100.0%	—	—
Married	2715	100.0%	—	—
Separated/divorced/widowed	803	100.0%	—	—

CI = confidence interval; IRSAD = Index of Relative Socio-economic Advantage and Disadvantage.

* Excludes 21 survey participants who did not report whether they had ever had an intimate partner and who were not asked questions about intimate partner violence.

† Proportions are weighted by age group, sex, Indigenous status, country of birth (Australia or overseas), highest educational level, and residential socio-economic status (Relative Socio-economic Advantage and Disadvantage quintiles).

‡ Not reported because of small cell numbers.

§ Fourteen participants of diverse genders not shown because of small cell numbers.

Table 3. Lifetime experience of intimate partner violence types among 7022 respondents with intimate partners at any time since age 16 years, overall and by age group and gender

		Any intimate partner violence		Physical violence		Sexual violence		Psychological violence	
**Age group**	**Respondents**	**Number**	**Proportion***	**Number**	**Proportion***	**Number**	**Proportion**	**Number**	**Proportion***
**All respondents**	**7022**	**3170**	**44.8% (43.3-46.2%)**	**1889**	**29.1% (27.7-30.4%)**	**925**	**11.7% (10.8-12.7%)**	**2897**	**41.2% (39.8-42.6%)**
Women	3558	1763	48.4% (46.3-50.4%)	1068	32.3% (30.3-34.2%)	711	18.2% (16.6-19.8%)	1631	45.1% (43.1-47.1%)
Men	3376	1345	40.4% (38.3-42.5%)	788	25.4% (23.5-27.2%)	175	4.0% (3.3-4.8%)	1216	36.6% (34.6-38.6%)
Diverse genders	88	62	69.1% (54.8-83.3%)	33	37.2% (23.3-51.2%)	39	42.0% (27.6-56.5%)	50	56.3% (41.5-71.0%)
**16-24 years**	**2201**	**1056**	**48.4% (46.1-50.6%)**	**530**	**25.2% (23.2-27.2%)**	**401**	**18.2% (16.5-20.0%)**	**964**	**44.2% (42.0-46.5%)**
Women	1099	576	52.5% (49.3-55.7%)	287	26.8% (24.0-29.6%)	286	25.8% (23.0-28.6%)	529	48.4% (45.2-51.6%)
Men	1048	442	42.8% (39.5-46.1%)	222	22.6% (19.7-25.5%)	89	8.5% (6.5-10.4%)	405	39.3% (36.0-42.5%)
**25-44 years**	**1868**	**928**	**51.4% (48.9-53.9%)**	**564**	**33.0% (30.6-35.4%)**	**252**	**13.9% (12.2-15.7%)**	**866**	**48.2% (45.7-50.7%)**
Women	945	499	53.8% (50.3-57.3%)	301	34.8% (31.4-38.2%)	185	20.8% (17.9-23.7%)	474	51.5% (47.9-55.0%)
Men	903	414	48.5% (44.8-52.1%)	257	31.0% (27.6-34.4%)	57	5.9% (4.3-7.5%)	378	44.4% (40.7-48.0%)
**45 years and over**	**2953**	**1186**	**39.9% (37.9-41.9%)**	**795**	**27.1% (25.3-28.9%)**	**272**	**9.3% (8.1-10.5%)**	**1067**	**36.2% (34.2-38.1%)**
Women	1514	688	44.3% (41.5-47.1%)	480	31.5% (28.8-34.1%)	240	15.5% (13.4-17.5%)	628	40.7% (37.9-43.4%)
Men	1425	489	34.6% (31.9-37.4%)	309	22.0% (19.6-24.4%)	29	2.1% (1.3-3.0%)	433	31.0% (28.3-33.7%)

* Proportions are weighted by age group, sex, Indigenous status, country of birth (Australia or overseas), highest educational level, and residential socio-economic status (Relative Socio-economic Advantage and Disadvantage quintiles).

Table 4. Summary of missing data for 8482 ACMS respondents who reported intimate partners since age 16 years, who they did not know or refused to answer individual questions about intimate partner violence*

	Number of respondents who reported they did not know or refused to answer the question	
**Did any partner ever ...**	**Number**	**Proportion**
blame you for causing their violent behaviour?	13	0.18%
shake, push, grab or throw you?	9	0.13%
try to convince your family, children or friends that you were crazy, or try to turn them against you?	23	0.32%
use or threaten to use a knife, gun or other weapon to harm you?	10	0.14%
make you perform sex acts you did not want to?	21	0.30%
follow you or hang around outside your home or work?	15	0.21%
threaten to harm or kill you or someone close to you?	9	0.13%
choke you?	16	0.23%
force or try to force you to have sex?	20	0.28%
harass you by phone, text, email or social media?	16	0.23%
tell you that you were crazy, stupid or not good enough?	20	0.28%
hit you with a fist or object, or kick or bite you?	19	0.27%
keep you from seeing or talking to your family or friends?	17	0.24%
confine or lock you in a room or other space?	15	0.21%
keep you from having access to a job, money or financial resources?	18	0.26%

* Respondents who reported that they did not know or refused to answer individual questions on intimate partner violence were treated as not having experienced that form of intimate partner violence for the purpose of this analysis.

Table 5. Lifetime experience of specific forms of physical intimate partner violence forms among 7022 respondents with intimate partners at any time since age 16 years, by age group and gender

	Any physical violence		Shake, push, grab or throw you		Use or threaten to use a knife, gun or other weapon to harm you	
**Age group**	**Number**	**Proportion***	**Number**	**Proportion***	**Number**	**Proportion***
**All respondents**	**1889**	**29.1% (27.7-30.4%)**	**1535**	**23.7% (22.5-25.0%)**	**519**	**8.8% (8.0-9.7%)**
Women	1068	32.3% (30.3-34.2%)	961	29.0% (27.2-30.9%)	300	10.1% (8.8-11.4%)
Men	788	25.4% (23.5-27.2%)	549	17.8% (16.2-19.5%)	214	7.5% (6.3-8.6%)
Diverse genders	33	37.2% (23.3-51.2%)	25	24.1% (12.6-35.5%)	5	6.1% (0.0-13.2%)
**16-24 years**	**530**	**25.2% (23.2-27.2%)**	**419**	**19.8% (18.0-21.6%)**	**120**	**5.9% (4.7-7.0%)**
Women	287	26.8% (24.0-29.6%)	254	23.9% (21.2-26.7%)	68	6.3% (4.8-7.8%)
Men	222	22.6% (19.7-25.5%)	148	14.5% (12.2-16.9%)	49	5.5% (3.8-7.2%)
**25-44 years**	**564**	**33.0% (30.6-35.4%)**	**482**	**28.1% (25.8-30.4%)**	**152**	**9.6% (8.0-11.1%)**
Women	301	34.8% (31.4-38.2%)	282	32.6% (29.3-36.0%)	87	11.2% (8.8-13.5%)
Men	257	31.0% (27.6-34.4%)	198	23.7% (20.5-26.8%)	64	8.0% (6.0-10.1%)
**45 years and over**	**795**	**27.1% (25.3-28.9%)**	**634**	**21.5% (19.8-23.1%)**	**247**	**8.8% (7.6-9.9%)**
Women	480	31.5% (28.8-34.1%)	425	27.6% (25.0-30.1%)	145	10.0% (8.2-11.7%)
Men	309	22.0% (19.6-24.4%)	203	14.4% (12.4-16.4%)	101	7.4% (5.9-8.9%)
	**Choke you**		**Hit you with a fist or object, or kick or bite you**		**Confine or lock you in a room or other space**	
**Age group**	**Number**	**Proportion**	**Number**	**Proportion**	**Number**	**Proportion**
**All respondents**	**515**	**7.9% (7.1-8.7%)**	**1183**	**19.3% (18.1-20.4%)**	**271**	**4.1% (3.5-4.7%)**
Women	383	11.6% (10.3-12.9%)	578	19.1% (17.4-20.8%)	210	6.5% (5.4-7.5%)
Men	123	3.8% (3.0-4.6%)	587	19.4% (17.7-21.1%)	53	1.4% (0.9-1.8%)
Diverse genders	9	10.6% (1.0-20.2%)	18	20.6% (9.4-31.7%)	8	8.6% (1.2-16.0%)
**16-24 years**	**162**	**7.6% (6.4-8.8%)**	**306**	**15.0% (13.3-16.7%)**	**86**	**4.4% (3.4-5.4%)**
Women	113	10.8% (8.8- 12.9%)	137	13.2% (11.0-15.4%)	61	5.9% (4.4-7.4%)
Men	43	4.0% (2.8- 5.2%)	158	16.5% (13.8-19.1%)	20	2.4% (1.1-3.8%)
**25-44 years**	**164**	**10.0% (8.4- 11.5%)**	**365**	**22.5% (20.4-24.7%)**	**82**	**5.1% (4.0-6.3%)**
Women	119	14.4% (11.8- 16.9%)	161	20.5% (17.5-23.5%)	66	8.5% (6.3-10.6%)
Men	43	5.2% (3.5- 6.9%)	201	24.8% (21.6- 28.0%)	14	1.5% (0.7-2.4%)
**45 years and over**	**189**	**6.6% (5.6- 7.6%)**	**512**	**17.8% (16.2-19.3%)**	**103**	**3.4% (2.7-4.1%)**
Women	151	10.0% (8.3-11.7%)	280	19.1% (16.8-21.4%)	83	5.3% (4.1-6.6%)
Men	37	2.8% (1.8-3.8%)	228	16.2% (14.1-18.3%)	19	1.1% (0.6-1.7%)

* Proportions are weighted by age group, sex, Indigenous status, country of birth (Australia or overseas), highest educational level, and residential socio-economic status (Relative Socio-economic Advantage and Disadvantage quintiles).

Table 6. Lifetime experience of specific forms of sexual intimate partner violence forms among 7022 respondents with intimate partners at any time since age 16 years, by age group and gender

	**Any sexual violence**		**Make you perform sex acts you did not want to**		**Force or try to force you to have sex**	
**Age group**	**Number**	**Proportion***	**Number**	**Proportion***	**Number**	**Proportion***
**All respondents**	**925**	**11.7% (10.8-12.7%)**	**720**	**9.0% (8.2-9.8%)**	**787**	**10.0% (9.1-10.9%)**
Women	711	18.2% (16.6-19.8%)	576	14.5% (13.1-15.9%)	608	15.6% (14.2-17.1%)
Men	175	4.0% (3.3-4.8%)	110	2.5% (1.9-3.1%)	148	3.4% (2.7-4.1%)
Diverse genders	39	42.0% (27.6-56.5%)	34	31.9% (18.8-44.9%)	31	32.9% (19.2-46.7%)
**16-24 years**	**401**	**18.2% (16.5-20.0%)**	**325**	**14.6% (13.0-16.2%)**	**338**	**15.5% (13.9-17.2%)**
Women	286	25.8% (23.0-28.6%)	244	22.0% (19.3-24.6%)	240	21.8% (19.2-24.4%)
Men	89	8.5% (6.5-10.4%)	57	5.2% (3.6-6.8%)	77	7.6% (5.7-9.5%)
**25-44 years**	**252**	**13.9% (12.2-15.7%)**	**198**	**11.2% (9.6-12.8%)**	**216**	**12.0% (10.3-13.6%)**
Women	185	20.8% (17.9-23.7%)	155	17.8% (15.1-20.6%)	162	18.3% (15.5-21.0%)
Men	57	5.9% (4.3-7.5%)	34	3.5% (2.3-4.8%)	47	4.8% (3.4-6.3%)
**45 years and over**	**272**	**9.3% (8.1-10.5%)**	**197**	**6.7% (5.7-7.7%)**	**233**	**7.9% (6.8-9.0%)**
Women	240	15.5% (13.4-17.5%)	177	11.3% (9.5-13.1%)	206	13.1% (11.2-15.0%)
Men	29	2.1% (1.3-3.0%)	19	1.4% (0.7-2.1%)	24	1.8% (1.0-2.6%)

* Proportions are weighted by age group, sex, Indigenous status, country of birth (Australia or overseas), highest educational level, and residential socio-economic status (Relative Socio-economic Advantage and Disadvantage quintiles).

Table 7. Lifetime experience of specific forms of psychological intimate partner violence forms among 7022 respondents with intimate partners at any time since age 16 years, by age group and gender

	Any psychological violence		Blame you for causing their violent behaviour?		Try to convince your family, children or friends that you were crazy, or try to turn them against you?		Follow you or hang around outside your home or work?		Threaten to harm or kill you or someone close to you?	
**Age group**	**Number**	**Proportion***	**Number**	**Proportion***	**Number**	**Proportion***	**Number**	**Proportion***	**Number**	**Proportion***
**All respondents**	**2897**	**41.2% (39.8-42.6%)**	**1467**	**21.7% (20.5-22.9%)**	**1148**	**16.9% (15.8-18.0%)**	**945**	**14.5% (13.5-15.5%)**	**660**	**11.1% (10.1-12.1%)**
Women	1631	45.1% (43.1-47.1%)	980	28.5% (26.6-30.3%)	644	18.6% (17.0-20.2%)	645	19.3% (17.6-20.9%)	453	15.0% (13.5-16.6%)
Men	1216	36.6% (34.6-38.6%)	458	14.1% (12.7-15.6%)	483	15.0% (13.5-16.5%)	288	9.3% (8.1-10.5%)	199	6.8% (5.7-7.9%)
Diverse genders	50	56.3% (41.5-71.0%)	29	28.3% (15.5-41.1%)	21	27.5% (14.2-40.8%)	12	11.4% (2.1-20.8%)	8	9.1% (0.0-18.3%)
**16-24 years**	**964**	**44.2% (42.0-46.5%)**	**467**	**22.3% (20.4-24.2%)**	**371**	**17.1% (15.4-18.8%)**	**250**	**11.5% (10.1-13.0%)**	**157**	**7.4% (6.2-8.7%)**
Women	529	48.4% (45.2-51.6%)	299	28.8% (25.9-31.8%)	198	17.8% (15.4-20.2%)	164	14.7% (12.4-16.9%)	103	9.3% (7.5-11.1%)
Men	405	39.3% (36.0-42.5%)	147	14.4% (12.1-16.7%)	161	16.1% (13.6-18.6%)	77	7.9% (6.0-9.9%)	48	5.3% (3.6-7.0%)
**25-44 years**	**866**	**48.2% (45.7-50.7%)**	**442**	**26.1% (23.8-28.3%)**	**314**	**19.0% (17.0-21.0%)**	**321**	**18.3% (16.4-20.3%)**	**211**	**13.1% (11.4-14.9%)**
Women	474	51.5% (47.9-55.0%)	290	33.5% (30.1-36.9%)	184	22.0% (19.0-25.0%)	223	24.9% (21.8-27.9%)	146	18.4% (15.6-21.3%)
Men	378	44.4% (40.7-48.0%)	145	17.9% (15.0-20.7%)	124	15.5% (12.8-18.2%)	95	11.4% (9.0-13.7%)	64	7.7% (5.7-9.6%)
**45 years and over**	**1067**	**36.2% (34.2-38.1%)**	**558**	**18.8% (17.2-20.4%)**	**463**	**15.6% (14.1-17.1%)**	**374**	**12.5% (11.1-13.8%)**	**292**	**10.3% (9.1-11.6%)**
Women	628	40.7% (37.9-43.4%)	391	25.3% (22.8-27.7%)	262	16.6% (14.5-18.6%)	258	16.4% (14.4-18.5%)	204	13.8% (11.8-15.8%)
Men	433	31.0% (28.3-33.7%)	166	11.6% (9.8-13.4%)	198	14.4% (12.4-16.4%)	116	8.1% (6.5-9.6%)	87	6.5% (5.0-7.9%)

(continued next page)

	Harass you by phone, text, email or social media?		Tell you that you were crazy, stupid or not good enough?		Keep you from seeing or talking to your family or friends		Keep you from having access to a job, money or financial resources	
**Age group**	**Number**	**Proportion***	**Number**	**Proportion***	**Number**	**Proportion***	**Number**	**Proportion***
**All respondents**	**1550**	**21.3% (20.1-22.5%)**	**2201**	**30.8% (29.5-32.1%)**	**1131**	**16.7% (15.6-17.8%)**	**448**	**8.2% (7.4-9.1%)**
Women	873	23.3% (21.6-25.1%)	1292	35.1% (33.2-37.1%)	649	18.7% (17.1-20.3%)	315	11.1% (9.7-12.4%)
Men	651	18.9% (17.3-20.6%)	870	25.8% (24.0-27.6%)	465	14.5% (13.0-16.0%)	129	5.2% (4.2-6.2%)
Diverse genders	26	26.6% (14.0-39.1%)	39	43.5% (29.0-58.0%)	17	16.2% (6.2-26.1%)	4	*a* (a)
**16-24 years**	**545**	**25.5% (23.5-27.5%)**	**758**	**34.7% (32.5-36.9%)**	**374**	**17.5% (15.8-19.3%)**	**70**	**3.2% (2.4-4.0%)**
Women	301	28.0% (25.1-30.9%)	434	39.3% (36.2-42.5%)	202	18.8% (16.3-21.4%)	49	4.5% (3.2-5.9%)
Men	228	22.8% (19.9-25.6%)	300	29.3% (26.2-32.3%)	160	15.9% (13.4-18.4%)	18	1.7% (0.9-2.5%)
**25-44 years**	**544**	**30.5% (28.2-32.8%)**	**649**	**36.3% (33.9-38.7%)**	**352**	**21.1% (19.0-23.2%)**	**149**	**9.6% (8.0-11.1%)**
Women	314	34.7% (31.3-38.1%)	372	40.7% (37.3-44.2%)	202	24.1% (21.0-27.2%)	104	13.4% (10.8-15.9%)
Men	222	26.0% (22.8-29.2%)	266	31.2% (27.8-34.6%)	147	18.1% (15.3-20.9%)	45	5.7% (4.0-7.5%)
**45 years and over**	**461**	**14.7% (13.3-16.0%)**	**794**	**26.6% (24.9-28.4%)**	**405**	**13.7% (12.3-15.0%)**	**229**	**8.2% (7.0-9.3%)**
Women	258	15.6% (13.6-17.6%)	486	31.0% (28.4-33.6%)	245	15.3% (13.3-17.3%)	162	10.6% (8.9-12.3%)
Men	201	13.6% (11.7-15.6%)	304	21.7% (19.3-24.0%)	158	11.9% (10.0-13.8%)	66	5.4% (4.0-6.8%)

* Proportions are weighted by age group, sex, Indigenous status, country of birth (Australia or overseas), highest educational level, and residential socio-economic status (Relative Socio-economic Advantage and Disadvantage quintiles).

Figure 1. Combinations of intimate sexual partner violence among 7022 respondents with intimate partners at any time since age 16 years (UpSet plot)*

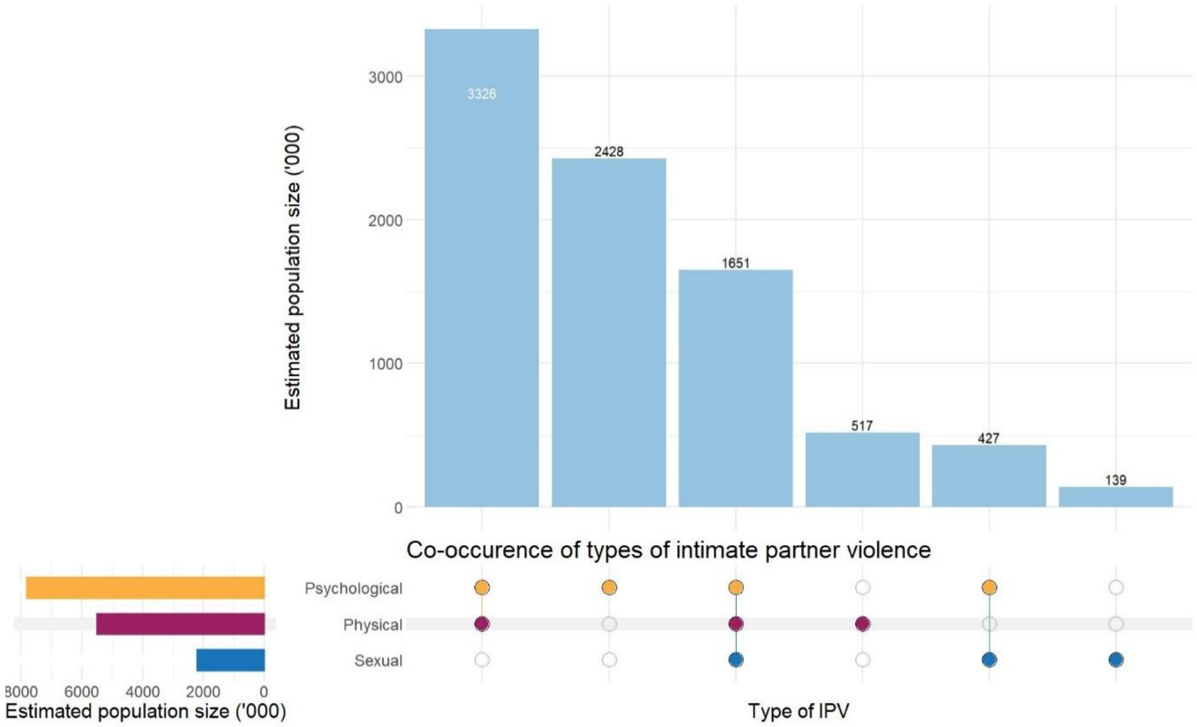

* The data in the graph is weighted (as per the axis labels). The 1651, for example, represents an estimated 1,651,000 Australian adults who have ever been in an intimate partner relationship who have experienced all three types of intimate partner violence.

Table 8. Lifetime experience of either physical or sexual intimate partner violence, or both, among 7022 respondents with intimate partners at any time since age 16 years, overall and by age group and gender

Age group	Respondents	Number	Proportion*
**All respondents**	**7022**	**2185**	**32.0% (30.7-33.4%)**
Women	3558	1291	36.7% (34.8-38.7%)
Men	3376	845	26.5% (24.6-28.3%)
Gender diverse	88	49	53.7% (38.9-68.5%)
**16-24 years**	**2201**	**692**	**32.2% (30.1-34.4%)**
Women	1099	405	37.1% (34.0-40.2%)
Men	1048	256	25.6% (22.6-28.6%)
**25-44 years**	**1868**	**643**	**36.8% (34.3-39.2%)**
Women	945	359	40.3% (36.9-43.8%)
Men	903	273	32.6% (29.2-36.1%)
**45 years and over**	**2953**	**850**	**28.9% (27.1-30.8%)**
Women	1514	527	34.4% (31.7-37.1%)
Men	1425	316	22.5% (20.1-24.9%)

* Proportions are weighted by age group, sex, Indigenous status, country of birth (Australia or overseas), highest educational level, and residential socio-economic status (Relative Socio-economic Advantage and Disadvantage quintiles).

References

1. Australian Bureau of Statistics. Australian Statistical Geography Standard (ASGS), edition 3, July 2021 - June 2026. 20 July 2021. https://www.abs.gov.au/statistics/standards/australian-statistical-geography-standard-asgs-edition-3/jul2021-jun2026 (viewed Apr 2025).

2. Australian Bureau of Statistics. Index of Relative Socio-economic Advantage and Disadvantage (IRSAD). In: Socio-Economic Indexes for Areas (SEIFA), Australia, 2021. 27 Apr 2023. https://www.abs.gov.au/statistics/people/people-and-communities/socio-economic-indexes-areas-seifa-australia/latest-release#index-of-relative-socio-economic-advantage-and-disadvantage-irsad- (viewed Apr 2025).

3. Haslam DM, Lawrence DM, Mathews B, et al. The Australian Child Maltreatment Study (ACMS), a national survey of the prevalence of child maltreatment and its correlates: methodology. Med J Aust 2023; 218 (6 Suppl): S5-S12.

Checklist for reporting of survey studies (CROSS)

Note: The numbers in this table refer to the submitted manuscript, not the published article or its Supporting Information file.

Section/topic	Item	Item description	Reported on page #
**Title and abstract**			
Title and abstract	1a	State the word "survey" along with a commonly used term in title or abstract to introduce the study's design.	1
	1b	Provide an informative summary in the abstract, covering background, objectives, methods, findings/results, interpretation/discussion, and conclusions.	1
**Introduction**			
Background	2	Provide a background about the rationale of study, what has been previously done, and why this survey is needed.	2-3
Purpose/aim	3	Identify specific purposes, aims, goals, or objectives of the study.	3
**Methods**			
Study design	4	Specify the study design in the methods section with a commonly used term (e.g., cross-sectional or longitudinal).	3
	5a	Describe the questionnaire (e.g., number of sections, number of questions, number and names of instruments used).	3-4
Data collection methods	5b	Describe all questionnaire instruments that were used in the survey to measure particular concepts. Report target population, reported validity and reliability information, scoring/classification procedure, and reference links (if any).	3-4
	5c	Provide information on pretesting of the questionnaire, if performed (in the article or in an online supplement). Report the method of pretesting, number of times questionnaire was pre-tested, number and demographics of participants used for pretesting, and the level of similarity of demographics between pre-testing participants and sample population.	n/a
	5d	Questionnaire if possible, should be fully provided (in the article, or as appendices or as an online supplement).	Provided in Supporting Information
Sample characteristics	6a	Describe the study population (i.e., background, locations, eligibility criteria for participant inclusion in survey, exclusion criteria).	3
	6b	Describe the sampling techniques used (e.g., single stage or multistage sampling, simple random sampling, stratified sampling, cluster sampling, convenience sampling). Specify the locations of sample participants whenever clustered sampling was applied.	3
	6c	Provide information on sample size, along with details of sample size calculation.	3
	6d	Describe how representative the sample is of the study population (or target population if possible), particularly for population-based surveys.	3-4
Survey administration	7a	Provide information on modes of questionnaire administration, including the type and number of contacts, the location where the survey was conducted (e.g., outpatient room or by use of online tools, such as SurveyMonkey).	3-4
	7b	Provide information of survey's time frame, such as periods of recruitment, exposure, and follow-up days.	3
	7c	Provide information on the entry process:<br>–>For non-web-based surveys, provide approaches to minimize human error in data entry.<br>–>For web-based surveys, provide approaches to prevent "multiple participation" of participants.	3

Study preparation	8	Describe any preparation process before conducting the survey (e.g., interviewers' training process, advertising the survey).	3
Ethical considerations	9a	Provide information on ethical approval for the survey if obtained, including informed consent, institutional review board [IRB] approval, Helsinki declaration, and good clinical practice [GCP] declaration (as appropriate).	4
	9b	Provide information about survey anonymity and confidentiality and describe what mechanisms were used to protect unauthorized access.	4
Statistical analysis	10a	Describe statistical methods and analytical approach. Report the statistical software that was used for data analysis.	4
	10b	Report any modification of variables used in the analysis, along with reference (if available).	n/a
	10c	Report details about how missing data was handled. Include rate of missing items, missing data mechanism (i.e., missing completely at random [MCAR], missing at random [MAR] or missing not at random [MNAR]) and methods used to deal with missing data (e.g., multiple imputation).	4
	10d	State how non-response error was addressed.	4-5
	10e	For longitudinal surveys, state how loss to follow-up was addressed.	n/a
	10f	Indicate whether any methods such as weighting of items or propensity scores have been used to adjust for non-representativeness of the sample.	3
	10g	Describe any sensitivity analysis conducted.	n/a
**Results**			
Respondent characteristics	11a	Report numbers of individuals at each stage of the study. Consider using a flow diagram, if possible.	4-6
	11b	Provide reasons for non-participation at each stage, if possible.	n/a
	11c	Report response rate, present the definition of response rate or the formula used to calculate response rate.	5
	11d	Provide information to define how unique visitors are determined. Report number of unique visitors along with relevant proportions (e.g., view proportion, participation proportion, completion proportion).	n/a
Descriptive results	12	Provide characteristics of study participants, as well as information on potential confounders and assessed outcomes.	Box 1
Main findings	13a	Give unadjusted estimates and, if applicable, confounder-adjusted estimates along with 95% confidence intervals and p-values.	5-6
	13b	For multivariable analysis, provide information on the model building process, model fit statistics, and model assumptions (as appropriate).	n/a
	13c	Provide details about any sensitivity analysis performed. If there are considerable amount of missing data, report sensitivity analyses comparing the results of complete cases with that of the imputed dataset (if possible).	n/a
**Discussion**			
Limitations	14	Discuss the limitations of the study, considering sources of potential biases and imprecisions, such as non-representativeness of sample, study design, important uncontrolled confounders.	8
Interpretations	15	Give a cautious overall interpretation of results, based on potential biases and imprecisions and suggest areas for future research.	6-7

Generalizability	16	Discuss the external validity of the results.	8
**Other sections**			
Role of funding source	17	State whether any funding organization has had any roles in the survey's design, implementation, and analysis.	Included in manuscript submission
Conflict of interest	18	Declare any potential conflict of interest.	Included in manuscript submission
Acknowledgements	19	Provide names of organizations/persons that are acknowledged along with their contribution to the research.	Included in manuscript submission
